# Relationships between Humor Styles and the Big Five Personality Traits in Workers: A Network Analysis

**DOI:** 10.3390/ijerph20021008

**Published:** 2023-01-05

**Authors:** Annamaria Di Fabio, Alessio Gori, Andrea Svicher

**Affiliations:** 1 Department of Education, Languages, Intercultures, Literatures and Psychology (Psychology Section), University of Florence, via di San Salvi, 12, Complesso di San Salvi, Padiglione 26, 50135 Florence, Italy; 2 Department of Health Sciences (Psychology Section), University of Florence, via di San Salvi, 12, Complesso di San Salvi, Padiglione 26, 50135 Florence, Italy

**Keywords:** Humor Styles Questionnaire, personality facets, psychometric network analysis, workplace

## Abstract

In this study, we investigated the relationship between the four humor styles (Affiliative, Self-enhancing, Aggressive, and Self-defeating) assessed via the Humor Styles Questionnaire (HSQ) and the ten facets of the Big Five Questionnaire (BFQ) through network analysis. Four hundred and sixty-two Italian workers (61.3% women; M_age_ = 48.59; SD = 10.75) participated in the study and filled out the HSQ and the BFQ. Both centrality indexes (Expected Influence [EI]) and bridge nodes were calculated. In addition, the stability and accuracy of the network were checked. The network analysis revealed that HSQ Self-enhancing (EI = 0.63) showed the highest centrality among the HSQ styles, whereas BFQ Emotion Control (EI = 1.10) showed the highest centrality among BFQ facets; it also revealed that they were positively linked. Furthermore, HSQ Self-defeating emerged as the second-most-central humor style, negatively associated with BFQ Emotion Control. Concerning Bridge dimensions, four nodes were identified: HSQ Aggressive Humor, BFQ Emotion Control, BFQ Dynamism, and BFQ Dominance, with positive links between humor and personality except for Aggressive humor and Emotion Control, which showed negative links. On the basis of these results, the high centrality of HSQ Self-enhancing indicates the possibility of using this node as a starting point to foster positive and adaptive humor styles. The centrality of HSQ Self-defeating suggests that strength-based interventions could be focused to increase adaptive humor styles and to decrease them in order to enhance health-promoting humor styles. Furthermore, the bridge node of the HSQ Aggressive humor style with specific personality facets shows its possible use in intervention to both resize and to adaptively improve relationships between humor and personality.

## 1. Introduction

The world of work in the 21st century is characterized by a growing technological acceleration of production and work processes [1], thus impacting the well-being of workers [2]. In this context, practices implementing workers’ well-being [3,4] have emerged. The positive strength-based approach [5,6,7,8] has considered the use of positive variables to build the psychological strength of workers and organizations, including the framework of humor [9,10].

Humor is a cognitive and emotional process that leads to perceiving, creating, understanding, and appreciating a stimulus (e.g., words, actions, films, pictures) as funny and tends to evoke the associated response of mirth involved in its enjoyment (e.g., amusement, exhilaration, smiling, laughter) [11]. Humor seems to be involved in most human behavior [12], and its function in the workplace has attracted the interest of applied psychologists for a century now [13,14]. The first studies in the field mainly emphasized the negative implications of humor; for example, describing the humorous remarks used by employees to express discomfort with challenging or very high workload) [15,16]. After that, since Malone [17] illustrated the case for and against humor in the workplace, researchers have explored the circumstance that humor may promote effectiveness in the workplace [18,19,20]. Results of subsequent studies highlighted an extensive array of links between humor and desirable organizational outcomes, such as enhanced work performance [21], organizational creativity [22,23], employee well-being [24,25], workgroup cohesion [26,27,28], and workers-to-workers communication [29]. A further advance stemmed from studies in psychoneuroimmunology, establishing that the sympathetic nervous system and the hypothalamus-pituitary-adrenal axis are activated by humor [30]. In this line, workers used positive humor as a coping mechanism [31,32] to mitigate the detrimental effects of workplace stress, decreasing burnout and work withdrawal [21]. While research has advanced the understanding of the construct in the working environment, humor defies a globally accepted definition, partly due to its multi-dimensional characteristics [29]. As a consequence, the literature reports a wide range of competing models from Craik and colleagues [33] that described five bipolar humorous styles (i.e., Socially Warm versus Cold; Competent versus Inept; Reflective versus Boorish; Earthy versus Repressed; Benign versus Mean-spirited) to Ruch et al. [34] that advanced eight comic styles (i.e., fun, humor, nonsense, wit, irony, satire, sarcasm, and cynicism). Furthermore, some other personality researchers have advanced another widely used model that focuses on distinguishing between four main types of humor styles that may be adaptive or maladaptive about the individual’s subjective well-being [32]. In this view, humor could have a double function involving pleasant and prosocial purposes (i.e., health-promoting) or hostile and malevolent intents (i.e., health-endangering) [32]. They are Affiliative, Self-enhancing, Aggressive, and Self-defeating humor styles [32]. Affiliative humor regards the use of pleasant banter to stimulating bonds between individuals. Self-enhancing humor reflects the capacity to perceive amusement amid life’s hardship. Aggressive humor involves the use of cynicism as well as humiliation to injure or manipulate others. Self-defeating humor deals with to one’s efforts to make other people laugh by derogatory and sarcastic remarks about oneself [32]. According to this conceptualization, Martin and colleagues created the Humor Styles Questionnaire (HSQ), which is the most applied instrument for measuring the humor styles [35]. The HSQ assessed the above-mentioned four humor styles, taking into account their possible impact on well-being [32].

### 1.1. Literature Review

In the workplace, health-promoting humor styles seem to favor workers’ well-being and positive organizational outcomes; in contrast, health-endangering styles seem to be negatively associated with well-being of workers and desirable work outcomes [21]. Furthermore, from a strengths-based perspective [8], health-promoting humor styles have emerged as promising dimensions [10] to fostering workers’ psychological resources and healthy organizational attitudes to overcome the challenges of the 21st-century world of work [1,2,36].

In this framework, several studies have also explored the links between the four humor styles and personality traits [35,37,38,39,40,41,42,43,44,45,46,47,48].

The most widely used personality model applied to investigate links between humor styles and personality is the “Big Five” [21,35,44]. It encompasses five main personality traits, namely Emotional Stability, Extraversion, Openness to Experience, Agreeableness, and Conscientiousness [49,50]. Accordingly, personality traits are sometimes represented as bipolar categories (Extraversion versus Introversion; Emotional stability versus Neuroticism; Agreeableness versus Antagonism; Conscientiousness versus Undependability; Openness to experience versus Closedness) [51] or labeled with diverse terms (Extraversion may be labeled Energy, Openness may be labeled Intellect). However, they are the same five dimensions [52].

Concerning the relationship between personality and humor styles, results from meta-analysis [35] and systematic review [44] showed that health-promoting humor styles (Affiliative and Self-enhancing styles) are positively linked with Extraversion, Agreeableness, Conscientiousness, and Openness, and negatively linked with Neuroticism. Contrastingly, health-endangering humor was positively related with Neuroticism and negatively related with Agreeableness and Conscientiousness [35,44].

During the last decade, the network approach [53] has started to be applied by personality researchers in the field of personality [54] and humor [43]. With regards to humor research, Lau et al. [43] firstly analyzed through network analysis an extensive array of variables related with the temperamental basis of humor enclosing those advanced by Martin et al. [32] and Ruch et al. [34]. This study highlighted three emotional dimensions as centerpieces of the temperamental basis of humor: cheerfulness, seriousness, and bad mood [43]. In turn, these dimensions were associated with two main clusters of humor-related personality traits [43]. The first one was labeled lighthearted humor, which was linked with cheerfulness. The second one was defined as humorlessness (e.g., gelotophobia, self-defeating humor, ineptness) and was associated with bad mood and seriousness [43]. Moreover, the network realm was increasingly used to explore the Big Five personality model [54,55] and its association with other personality-related constructs, such as adult temperament styles [56] and perfectionism [57,58], as well as for crucial variables in a health-promoting perspective in relation to the workplace, meaning at work [59], and decent work [60]. However, to our knowledge, the relationship between the four humor styles and the Big Five personality traits has not yet been investigated through the network approach in workers.

### 1.2. Aims of the Study

Therefore, the present study sought to analyze the relationships between the four humor styles and the Big Five personality traits in workers via a network approach. Different from the factor approach, the network realm conceives the structural covariance among humor styles and personality traits as not constrained in an a priori factor structure, but rather arising from reciprocal interactions among them. As a result, the network of humor styles and personality is an “ecosystem” in which some dimensions activate while others inhibit the ecosystem [54]. Accordingly, the network ecosystem of humor styles and personality traits comprises nodes and edges. Nodes reflect the humor dimensions and personality dimensions. Edges describe the association between nodes. In this approach, it is possible to determine the most central nodes and bridge nodes. Most central nodes are humor and personality dimensions that function as the centerpieces of the network. Bridge nodes are the most connected nodes in the network that bring together the two constructs (i.e., humor style and personality) [61]. Thus, the network approach was selected to identify the most central nodes as well as the bridge nodes considering a network enclosing humor styles and personality. As conceived by Martin et al. [32], humor styles can be classified through a 2 × 2 matrix in which health-promoting styles encompass Affiliative and Self-enhancing humor, whereas health-endangering styles include Aggressive and Self-defeating humor. Moreover, one advantage of the Big Five is that the model allows for more fine-grained analysis, using ten lower-level and more specific dimensions (facets) that compose the five broad dimensions. They are Dynamism, Dominance, Cooperativeness, Politeness, Scrupulousness, Perseverance, Emotion Control, Impulse Control, Openness to Culture, and Openness to Experiences [62].

Hence, the four humor styles and the ten facets of Big Five were entered as nodes of the network. This perspective could offer a contribution to better understand the relationships among humor styles and Big Five personality traits in workers that participated in our study.

## 2. Methods

### 2.1. Participants

Participants of this study were 462 workers employed in various public and private organizations in central-southern Italy (61.3% women; Mean Age = 48.59; SD = 10.75; 61.7% had at least a high school education). Confidentiality was guaranteed and participation was voluntary. Each participant signed a privacy protection disclaimer in accordance with Italian law’s standard criteria for ethics in research (Law Decree DL-196/2003) and European Union General Data Protection Regulation (EU 2016/679).

### 2.2. Humor Styles Questionnaire

The Humor Styles Questionnaire (HSQ) [32]—Italian version [9] is a is a 32-item self-report questionnaire with items rated on a 7-point Likert scale ranked between 1 (totally disagree) and 7 (totally agree). The HSQ measures for humor styles: Affiliative humor, Self-enhancing humor, Aggressive humor, and Self-defeating humor [9,32]. Internal consistency assessed through Cronbach’s alpha was found to vary from 0.70 (HSQ Self-enhancing) to 0.80 (HSQ Affiliative). Internal consistency measured via McDonald’s Omega ranged from 0.71 (HSQ Self-enhancing) to 0.82 (HSQ Affiliative) (Table 1).

### 2.3. Big Five Questionnaire

The Big Five Questionnaire (BFQ) [62] is a 132-item questionnaire that measures the Five factor model of personality (Extraversion, Agreeableness, Conscientiousness, Emotional Stability, and Openness) on a 5-point Likert scale from 1 (absolutely false) to 5 (absolutely true). Furthermore, each five personality traits have two facets, for a total of ten facets [62]. Extraversion consists of Dynamism and Dominance. Agreeableness is composed of Cooperativeness and Politeness. Conscientiousness encloses Scrupulousness and Perseverance. Emotional Stability encompasses Emotion Control and Impulse Control. Openness includes Openness to Culture and Openness to Experiences [62]. In the current study, values of Cronbach’s alpha were found to be from 0.71 (Openness to Culture) to 0.93 (Emotion Control), whereas McDonald’s Omega coefficients ranged from 0.73 (Openness to Culture) to 0.94 (Emotion Control) (Table 1).

### 2.4. Statistical Analysis

We estimated zero-order correlations and after the network model. Before proceeding with network analyses, we examined each study variable’s mean, standard deviation, kurtosis, and skewness. Subsequently, we estimate a network structure (lambda tuning = 0.001) [63] enclosing the four HSQ dimensions and ten BFQ facets by following Burger et al.’s guidelines [64]. Our network model comprises fourteen nodes (four reflecting the HSQ humor styles whereas ten mirroring the BFQ facets) and edges representing the regularized partial correlation between two nodes (i.e., controlled by all surrounding ones). In this model, edges in blue color displayed positive associations, whereas edges in red displayed negative associations. The magnitude of each node is represented by its thickness (nodes that are thicker reflect a higher value) [53]. The R packages (R Foundation for Statistical Computing, Vienna, Austria) bootnet 1.5 and qgraph 1.9 were used.

Expected Influence index (EI) was implemented to assess local network properties [65]. The EI index is a superior centrality index that calculates each node’s overall connections [65]. Node predictability (ranging from 0 to 1) was also evaluated. It displays how a particular node is predicted by all surrounding nodes, illustrating the percentage of variance shared by a specific node with all neighboring nodes [66]. The correlation stability (CS) coefficient was used to calculate network stability (CS coefficient > 0.50 suggests a stable EI) [66]. The nonparametric bootstrapped difference test for EI was applied to inspect differences in EI that were statistically significant. The nonparametric bootstrapped difference test for edge weight was applied to examine the differences between edges that were statistically significant [66]. The bootstrap test of edge weight accuracy was used to examine network accuracy [66]. Finally, we calculated the bridge Expected Influence to identify bridge nodes that connected the investigated constructs (humor style assessed via HSQ and personality facets measured through BFQ). Following Jones et al. [61], we implemented a graphical LASSO model. It displays the nodes with bridge EI in the top 80% of the percentiles’ distribution. We used the following R packages (R Foundation for Statistical Computing, Vienna, Austria): bootnet 1.5, networktools 1.2.3, and qgraph 1.9. All the analyses were conducted using the R Studio Version 2022.07.0 Build 548 (Posit Software, Boston, MA, USA) for Windows.

## 3. Results

**Figure 1:**
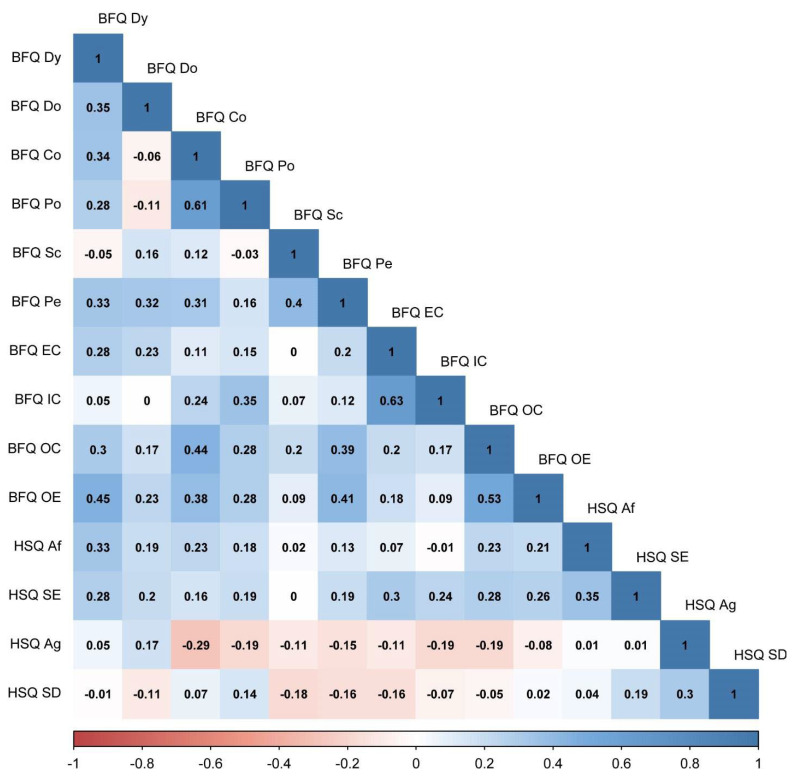
Zero order Pearson correlations (*n* = 462). Note: Big Five Questionnaire (BFQ) ten facets. Dy: Dynamism; Do: Dominance; Co: Cooperativeness; Po: Politeness; Sc: Scrupulousness; Pe: Perseverance; EC: Emotion Control; IC: Impulse Control; OC: Openness to Culture; OE: Openness to Experiences. Humor Style Questionnaire (HSQ) four dimensions. Af: Affiliative; SE: Self-enhancing; Ag: Aggressive; SD: Self-defeating.

**Figure 2:**
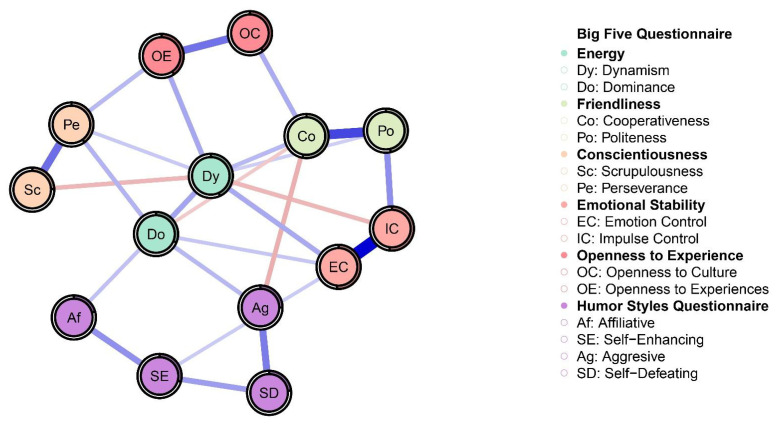
Network model for Humor Styles dimensions and Big Five Personality facets: Graphical representation (*n* = 462). Note: In the network model, the Humor Style Questionnaire dimensions and the ten facets of the Big Five Questionnaire are represented by nodes. Links between nodes are displayed via edges. Positive connections are shown by blue edges, whereas negative connections are shown by red edges; the stronger the connection, the thicker the edge. The proportion of shared variation of each node with its surrounding nodes is shown as a pie chart around the nodes.

**Figure 3:**
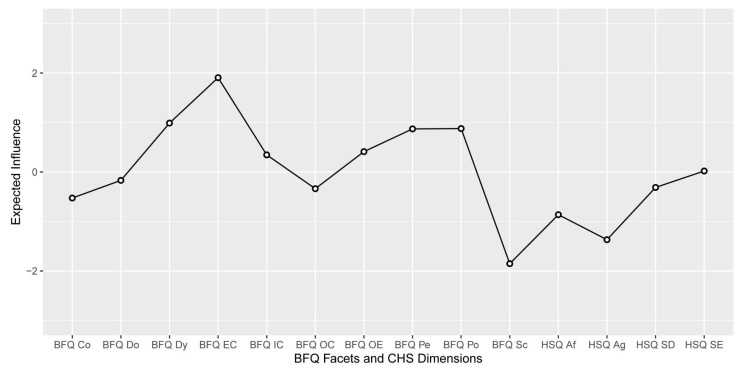
Network of Humor Styles dimensions and Big Five Personality facets: Expected influence centrality estimates (*n* = 462). Note: The Humor Style Questionnaire dimensions and Big Five Questionnaire personality traits are listed on the X-axis. Expected Influence as standardized z-score are reported on the Y-axis. Big Five Questionnaire (BFQ) ten facets. Dy: Dynamism; Do: Dominance; Co: Cooperativeness; Po: Politeness; Sc: Scrupulousness; Pe: Perseverance; EC: Emotion Control; IC: Impulse Control; OC: Openness to Culture; OE: Openness to Experiences. Humor Style Questionnaire (HSQ) four dimensions. Af: Affiliative; SE: Self-enhancing; Ag: Aggressive; SD: Self-defeating.

**Figure 4:**
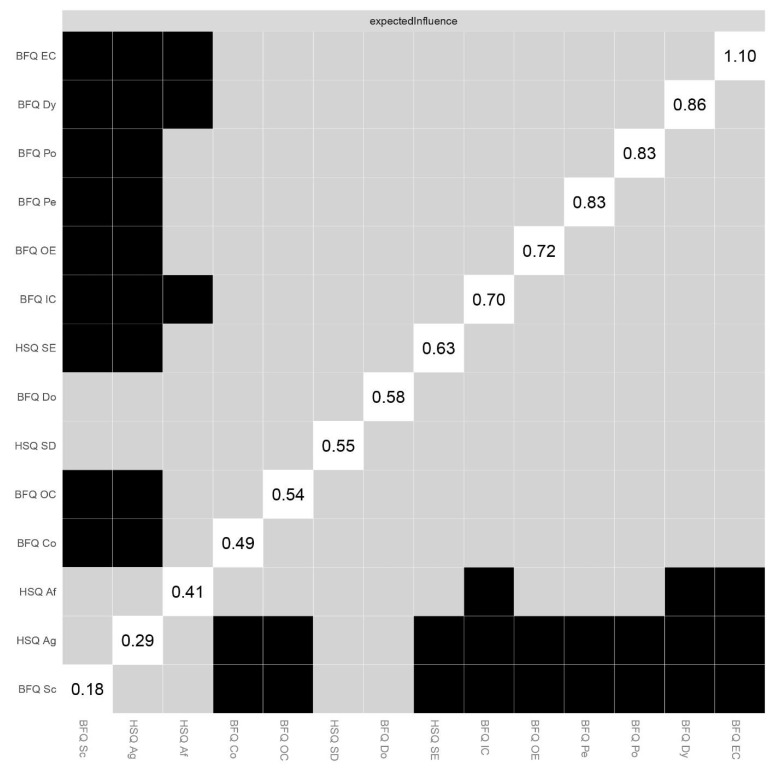
Network of Humor Styles dimensions and Big Five Personality facets (*n* = 462): Nonparametric bootstrapped difference test. Note: The Humor Style Questionnaire dimensions and Big Five Questionnaire personality facets are displayed on the X and Y axis. Numbers in withe boxes on diagonal illustrate Expected Influence (EI) centrality estimates (raw score). Black boxes represent a statistical difference between EIs, whereas gray boxes highlight a difference that is not statistically significant. Big Five Questionnaire (BFQ) ten facets. Dy: Dynamism; Do: Dominance; Co: Cooperativeness; Po: Politeness; Sc: Scrupulousness; Pe: Perseverance; EC: Emotion Control; IC: Impulse Control; OC: Openness to Culture; OE: Openness to Experiences. Humor Style Questionnaire (HSQ) four dimensions Af: Affiliative; SE: Self-enhancing; Ag: Aggressive; SD: Self-defeating.

**Figure 5:**
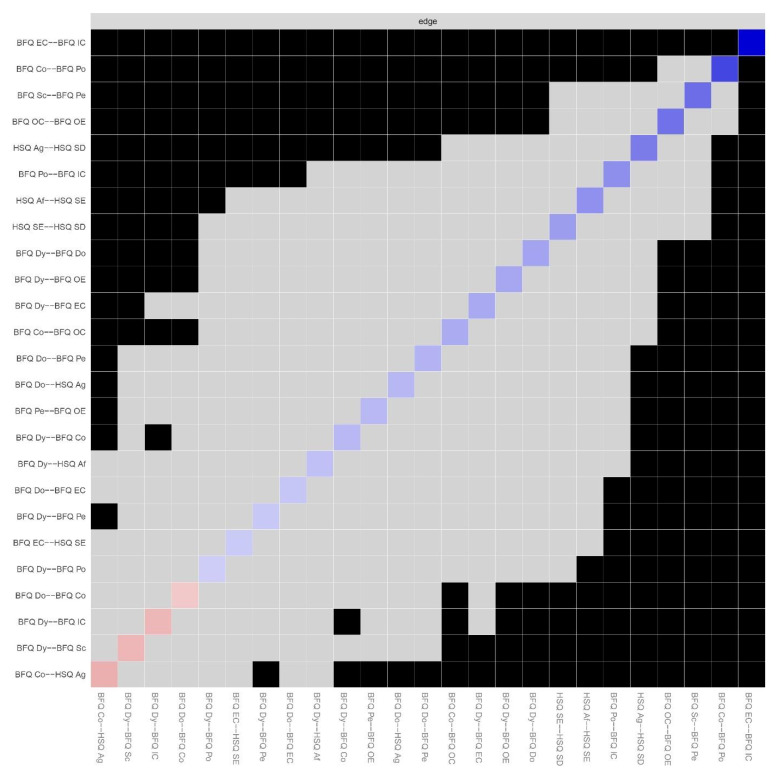
Network of Humor Styles dimensions and Big Five Personality facets: Nonparametric bootstrapped difference test (*n* = 462). Note: In ascending order, the X and Y axes indicate all edges in the network with non-zero values. Black boxes represent a statistical difference between two edge weights; gray boxes highlight a difference that is not statistically significant. Blue boxes in the diagonal illustrate positive edges; red boxes illustrate negative edges. A more intense color indicates a stronger edge. On the side of each box, labels display the two nodes connected via the analyzed edge. Big Five Questionnaire (BFQ) ten facets. Dy: Dynamism; Do: Dominance; Co: Cooperativeness; Po: Politeness; Sc: Scrupulousness; Pe: Perseverance; EC: Emotion Control; IC: Impulse Control; OC: Openness to Culture; OE: Openness to Experiences. Humor Style Questionnaire (HSQ) four dimensions. Af: Affiliative; SE: Self-enhancing; Ag: Aggressive; SD: Self-defeating.

Table 1 shows the means, standard deviations, skewness, and kurtosis of variables involved in the present research. Figure 1 reports Pearson correlations among study variables. Skewness and kurtosis for all variables were found ranging from 1 to −1, confirming that they were within acceptable parameters, highlighting no departures from normality. Thus, we run network analysis. Figure 2 displays the Network model of HSQ humor styles and BFQ personality facets. Figure 3 reports the Expected Influence (EI) (i.e., centrality index) for each node in the network. Concerning the HSQ humor styles, Self-enhancing showed the highest centrality (EI = 0.63), and the second most central nodes was HSQ Self-defeating (EI = 0.55). Regarding the BFQ ten facets, Emotion Control showed the highest centrality (EI = 1.10) (Figure 4). Concerning association between most central HSQ and BFQ nodes, HSQ Self-enhancing was positively linked with BFQ Emotion Control, whereas HSQ Self-defeating was negatively linked with BFQ Emotion Control (Figure 1). However, Figure 5 highlights the statistically significant and strongest edges, highlighting that the most prominent edges were those linking dimensions of the same construct.

**Figure 6:**
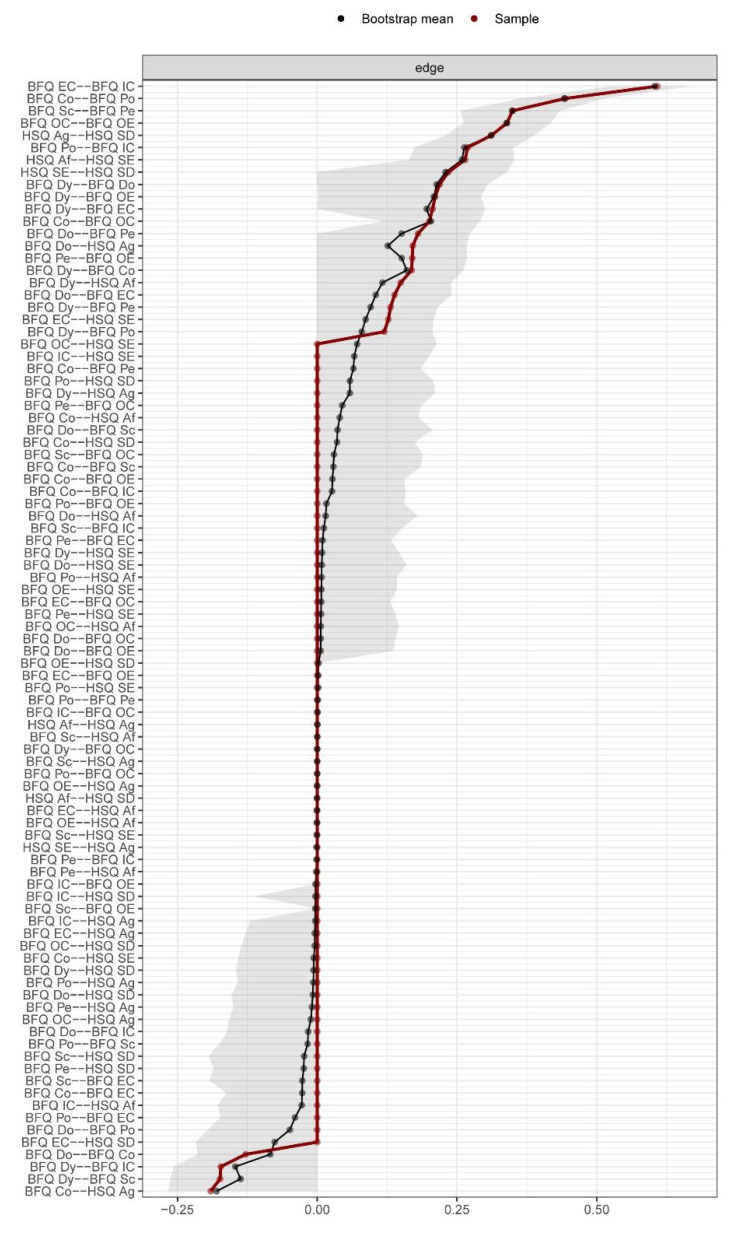
Network of Humor Styles dimensions and Big Five Personality facets: Bootstrap tests of the edge weight accuracy [95% confidence intervals] (*n* = 462). Note: Big Five Questionnaire (BFQ) ten facets. Dy: Dynamism; Do: Dominance; Co: Cooperativeness; Po: Politeness; Sc: Scrupulousness; Pe: Perseverance; EC: Emotion Control; IC: Impulse Control; OC: Openness to Culture; OE: Openness to Experiences. Humor Style Questionnaire (HSQ) four dimensions. Af: Affiliative; SE: Self-enhancing; Ag: Aggressive; SD: Self-defeating.

Mean node predictability was 0.51. It indicates that surrounding nodes could account for 51% of the variance of nodes. It ranged from 0.57 (BFQ Emotion Control) to 0.22 (HSQ Affiliative style). The CS coefficient was found to be high, with a value of 0.66, indicating the good trustworthiness of the network. Figure 6 shows the bootstrap tests of the edge weight accuracy. It displays a satisfactory level of precision for the 14 nodes that compose the network (Figure 6). Furthermore, the correlation between EI and predictability had a very high value (r_s_ = 0.87; *p* < 0.001), indicating that the EI was adequately stable. Lastly, EI and Node Predictability showed no statistically significant correlations with the mean levels of study’s variables (r_s_ = 0.16 [*p* = 0.408] and r_s_ = 0.17 [*p* = 0.415], respectively). In other words, strength (EI) and predictability are not associated with the mean scores of variables in the analyzed network.

**Figure 7:**
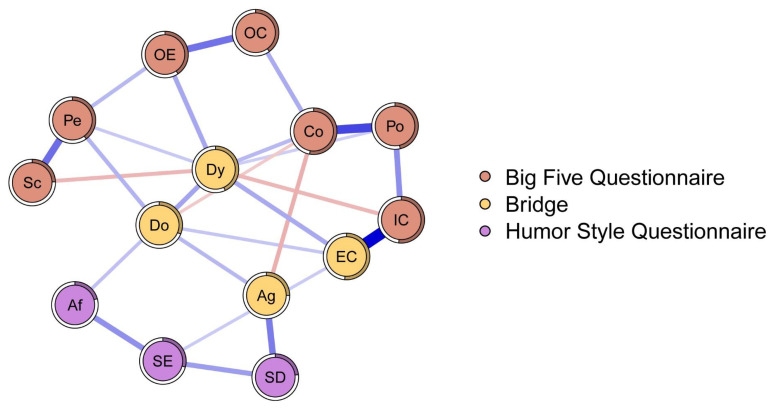
Network model of Humor Styles dimensions and Big Five Personality Facets: Graphical representation of the Bridge Nodes and Bridge Expected Influence *(n* = 462). Note: Big Five Questionnaire (BFQ) ten facets. Dy: Dynamism; Do: Dominance; Co: Cooperativeness; Po: Politeness; Sc: Scrupulousness; Pe: Perseverance; EC: Emotion Control; IC: Impulse Control; OC: Openness to Culture; OE: Openness to Experiences. Humor Style Questionnaire (HSQ) four dimensions. Af: Affiliative; SE: Self-enhancing; Ag: Aggressive; SD: Self-defeating.

Figure 7 illustrates the result regarding bridge nodes for the network composed of HSQ and BFQ dimensions. Results highlighted four bridge nodes for the analyzed network (Figure 7): HSQ Aggressive style, BFQ Emotion Control, BFQ Dynamism, and BFQ Dominance.

## 4. Discussion

To the best of our knowledge, the present study is the first research applying network analysis to investigate the relationships between humor styles and Big Five personality facets. Two primary findings have emerged and thus need to be discussed. The first finding is inherent to the degree of centrality observed in the network. The second finding deals with the bridge nodes that connected the two constructs (HSQ Humor styles and Big Five personality facets).

Concerning the degree of centrality, HSQ Self-enhancing emerged as the most central humor style among the HSQ styles, whereas BFQ Emotion Control emerged as the most central personality facet among BFQ facets. It indicates that the two dimensions are central in activating the network ecosystem of humor styles and personality facets. These results could suggest that participants from our study could be more prone to activated Self-enhancing humor style (the propensity to view life humorously, maintaining it even in the face of stressful circumstances or adversity) in their work environment [32]. Furthermore, workers from our study seemed to be characterized in their work environment by activating Emotion Control processes, which refer to the ability to manage emotionality and anxiety appropriately [62]. The link between the two most central HSQ and BFQ nodes was positive, in line with previous findings [35,42,45]. Furthermore, network analysis highlighted that HSQ Self-defeating (excessively self-disparaging humor, characterized by attempts to amuse others at the cost of and disadvantage to the self) was the second-most-central humor style among the HSQ styles. It was negatively associated with BFQ Emotion Control, in line with the literature [35,42,45]. This could suggest that the Self-enhancing humor style could be activated more often when Emotion Control is high. However, the Self-defeating humor style could be active when levels of Emotion Control are low, putting workers at risk of an active Self-defeating humor style.

Concerning the bridge nodes that connected the two constructs, the following four bridge nodes emerged: HSQ Aggressive humor, BFQ Emotion Control, BFQ Dominance, and BFQ Dynamism, showing positive links, except for HSQ Aggressive humor and BFQ Emotion Control, which showed a negative link. These findings could corroborate the idea that, in workers from our study, the Aggressive humor style (using sarcasm, teasing, ridicule, derision, “put-down”, or disparagement towards others, without consideration for the consequences or to manipulate others) could be more likely to occur when Emotion Control is low. Furthermore, network analysis showed that the Aggressive humor style had bridge functions with BFQ Dominance (being assertive and confident) and BFQ Dynamism (expansiveness and enthusiasm), both facets that pertain to extroversion. Though this is in contrast with previous results [35,38,44], it could be explained by the fact that our participants could reveal a vulnerability, expressing Aggressive humor style in association with high levels of Extraversion.

Strengths and weaknesses are present in the current study. The main strength is that links between humor styles and personality were examined for the first time by implementing network analysis [54]. Our results expand previous results obtained via the factor approach, highlighting main paths across humor styles and personality facets, considering the centrality of nodes, and identifying bridge nodes. In this view, most central and bridge nodes might reflect certain styles of humor that should be carefully analyzed and evaluated. The emergence of the Self-enhancing humor style as the most central HSQ dimension could indicate that the Self-enhancing humor style could be a suitable starting point for interventions aimed at strengthening the adaptive humor in our participants [8,10,67]. Furthermore, since the Self-defeating humor style was the second HSQ most central node, this finding could suggest that strength-based interventions could be focused to increase adaptive humor styles and to decrease Self-defeating humor to explore the learning of health-promoting humor styles [10,67]. The emergence of Aggressive humor as a bridge node in the participants in our study suggests the need to target this humor style in order to decrease maladaptive humor styles, both resizing it and at the same time acquiring more competences concerning health-promoting humor styles [10,67]. Lastly, since the Aggressive humor style was found to have a positive bridge function with both facets of Extraversion, an intervention focused on fostering more adaptive humor styles associated with BFQ Dominance (being assertive and confident) and BFQ Dynamism (expansiveness and enthusiasm) seems relevant to consider in relation to our participants [10,67].

With regard to limitations, our study has a cross-sectional design, so edges did not indicate whether a particular node causes or is caused by its neighboring node. In order to better understand the causal relationship, future research should apply longitudinal methods to study the network of humor styles and personality. Additionally, our study participants were Italian employees. Thus, future studies must be broadened to involve other countries as well as different cultural contexts.

## 5. Conclusions

Network analysis identified most central nodes as well as bridge nodes, thus suggesting evidence that could expand previous findings on the relationships between humor style and personality in the work environment [10]. It could be useful for trying to answer the recent call in the literature regarding how trait and personality theories interact with humor theories [14]. In our study run on workers, the most central HSQ node was Self-enhancing humor, also found positively associated with Emotion Control. This result on one side highlighted the centrality of this humor style in terms of a positive resource for workers, since the Self-enhancing humor style is positively related to well-being [14,68,69]. On the other side, this positive link between Self-enhancing humor and Emotion Control could highlight a potential aspect of strength of workers regarding our participants, in relation to this inwardly adaptive style, considering interactions between personality theories and humor theories [14]. Self-defeating humor was the second most central HSQ node, also negatively associated with Emotion Control. This result on one side could highlight the centrality of this humor style in terms of a potential aspect of vulnerability in workers from our study, since Self-defeating humor can be detrimental to workers’ well-being [68,69,70]. On the other side, considering interactions between personality theories and humor theories, this negative link between Self-defeating humor and Emotion Control in workers from our study could highlight a potential aspect of additional vulnerability, in relation to this inwardly maladaptive style [32]. With regards to bridge nodes, the Aggressive humor style had a bridge function with BFQ Dominance and BFQ Dynamism. This finding could highlight two potential paths of vulnerability in workers from our study. The first path of vulnerability is represented by the bridge function between Aggressive humor and BFQ Dominance which encompasses the ability to assert oneself, stand out, and influence others [32]. It could highlight a risk for workers from our study, since they could be more prone to use the Aggressive humor style together with BFQ Dominance facets at the workplace, perhaps increasing the likelihood of the use of this inwardly maladaptive humor style [70,71,72]. In the same way, our results highlighted a bridge between the Aggressive humor style and BFQ Dynamism (energy and enthusiasm), highlighting another path of risk for workers from our study. They could be more to prone use the Aggressive humor style associated with facets of Dynamism, perhaps increasing the likelihood of the use of this inwardly maladaptive humor style [70,71,72]. Thus, the application of network analysis could expand the knowledge on the relationship between stable individual characteristics of workers, such as personality, and those that are more malleable, such as humor styles. It could be insightful for approaches focused on enhancing positive resources for workers’ well-being [6,7,8,73] and healthy business, building healthy organizations [36]. In this light, identifying specific variables for research and interventions amidst malleable individual styles, such as humor, could be an advantage to plan further advancements in studying and fostering positive organizational outcomes [36].

In brief, our results expand on previous findings [35,44], suggesting that workers from our study activated the Self-enhancing humor style in association with BFQ Emotion control. Furthermore, the Aggressive humor style was found to have a negative bridge function with Emotion Control and a positive bridge function with Extraversion (BFQ Dominance and Dynamism).

Future research could expand the knowledge about the network ecosystem of humor styles and personality, enclosing variables focused on coping strategies associated with humor [31], the temperamental basis of humor [43], and emotional intelligence [74]. Even though further research is required, the network realm seems to be a promising approach to undercovering the key elements involved in the relationship between humor styles and personality traits in workers. In turn, identifying central and bridge aspects of humor styles in workers could serve as an indication to help researchers and practitioners to expand both research and intervention to use humor as a positive resource for enhancing the well-being of workers and organizations [2,3,8,57].

## Figures and Tables

**Table 1:** Humor Styles Questionnaire and Big Five Questionnaire: Means, Standard Deviations, Skewness, and Kurtosis, Cronbach’s alpha, and McDonald’s Omega (*n* = 462).

Study Variables	M	SD	Min	Max	Sk.	Kr.	α	ω
BFQ Dynamism	40.49	5.19	24	54	0.05	0.25	0.74	0.76
BFQ Dominance	34.61	5.78	17	53	0.17	0.70	0.77	0.80
BFQ Cooperativeness	41.98	5.46	22	58	−0.05	−0.02	0.78	0.79
BFQ Politeness	38.49	6.00	19	60	−0.16	0.37	0.77	0.81
BFQ Scrupulousness	40.30	6.85	16	59	−0.15	0.12	0.86	0.88
BFQ Perseverance	43.34	6.22	21	60	−0.24	0.45	0.88	0.91
BFQ Emotion Control	36.16	7.82	16	59	−0.05	−0.19	0.93	0.94
BFQ Impulse Control	35.60	7.00	12	58	−0.26	0.61	0.89	0.90
BFQ Openness to Culture	41.47	5.96	18	56	−0.25	0.03	0.71	0.73
BFQ Openness to Experiences	40.77	5.75	20	55	0.08	−0.17	0.86	0.87
HSQ Affiliative	40.43	8.43	9	56	−0.32	−0.03	0.80	0.82
HSQ Self-enhancing	35.16	7.58	16	56	0.10	−0.18	0.70	0.71
HSQ Aggressive	23.04	6.74	8	44	0.08	−0.56	0.71	0.73
HSQ Self-defeating	25.69	8.43	8	56	0.40	0.35	0.76	0.77

BFQ = Big Five Questionnaire; HSQ = Humor Styles Questionnaire; α = Cronbach’s alpha; ω = McDonald’s Omega.

## Data Availability

The data presented in this study are available from the corresponding author upon reasonable request. The data are not publicly available due to privacy reasons.
